# Supplementary material for: A Functional 67-bp Duplication Locating at the Core Promoter Region within the Bovine ADIPOQ Gene Is Associated with Ovarian Traits and mRNA Expression
Source: Animals (Basel). 2024 Aug 15;14(16):2362. doi: 10.3390/ani14162362 (PMC11350689; doi:10.3390/ani14162362)
Supplement: Supplementary file 1 [file animals-14-02362-s001.zip › animals-3146902-supplementary.pdf]

Supplementary Table

Supplementary Table 1 Relationship between the 67-bp duplication variation within the *ADIPOQ* gene and the ovarian traits(pre-oestrus and estrus) of Chinese Holstein (LSM±SE)

Oestrus cycle	Quantitative Traits		Genotypes		*P*-value
			DD	ID	
Pre-oestrus	Ovary	length (mm)	40.2±0.5(n=355)	41.0±2.4(n=4)	0.854
		width (mm)	15.8±0.2(n=355)	15.0±1.4(n=4)	0.729
		height (mm)	20.1±0.3(n=355)	18.5±1.8(n=4)	0.592
		weight (g)	6.4±0.2(n=356)	5.4±0.5(n=4)	0.534
	Mature follicle	number	0.0±0.0(n=282)	0.0±0.0(n=3)	0.811
		diameter (mm)	0.2±0.1(n=230)	0.0±0.0(n=3)	0.781
	Corpus luteum	number	0.0±0.0(n=248)	0.0±0.0(n=3)	0.913
		diameter (mm)	0.1±0.1(n=282)	0.0±0.0(n=3)	0.892
	Corpus albicans	number	0.7±0.0(n=286)	1.0±0.7(n=4)	0.406
		diameter (mm)	2.7±0.2(n=286)	2.0±1.2(n=4)	0.685
Estrus	Ovary	length (mm)	38.6±0.5(n=241)	31.6±3.5(n=5)	0.057
		width (mm)	18.3±0.3(n=241)	17.2±3.1(n=5)	0.633
		height (mm)	21.5±0.3(n=241)	19.4±1.5(n=5)	0.403
		weight (g)	7.9±0.2(n=239)	5.1±1.4(n=5)	0.077
	Mature follicle	number	1.3±0.0(n=241)	1.2±0.2(n=5)	0.818
		diameter (mm)	12.4±0.3(n=219)	14.2±3.0(n=5)	0.357
	Corpus luteum	number	0.0±0.0(n=128)	-	-
		diameter (mm)	0.0±0.0(n=111)	-	-
	Corpus albicans	number	0.6±0.1(n=142)	-	-
		diameter (mm)	2.7±0.3(n=141)	-	-

Note: Genotypes are omitted if the number of individuals is less than 3.
